# High throughput sequencing of *in vitro* selections of mRNA-displayed peptides: data analysis and applications

**DOI:** 10.1039/c9cp05912a

**Published:** 2020-01-22

**Authors:** Celia Blanco, Sam Verbanic, Burckhard Seelig, Irene A. Chen

**Affiliations:** 1. Department of Chemistry and Biochemistry 9510, University of California, Santa Barbara, CA 93106; 2. Department of Chemical and Biomolecular Engineering, University of California, Los Angeles, CA 90095; 3. Program in Biomolecular Sciences and Engineering, University of California, Santa Barbara, CA 93106; 4. Department of Biochemistry, Molecular Biology and Biophysics, University of Minnesota, Minneapolis, MN, 55455 USA; 5. BioTechnology Institute, University of Minnesota, St. Paul, MN, 55108, USA

**Keywords:** mRNA display, high throughput sequencing, molecular evolution, directed evolution

## Abstract

*In vitro* selection using mRNA display is currently a widely used method to isolate functional peptides with desired properties. The analysis of High Throughput Sequencing (HTS) data from *in vitro* evolution experiments has proven to be a powerful technique but only recently has it been applied to mRNA display selections. In this Perspective, we introduce aspects of mRNA display and HTS that may be of interest to physical chemists. We highlight the potential of HTS to analyze *in vitro* selections of peptides and review recent advances in the application of HTS analysis to mRNA display experiments. We discuss some possible issues involved with HTS analysis and summarize some strategies to alleviate them. Finally, the potential for future impact of advancing HTS analysis on mRNA display experiments is discussed.

## Graphical Abstract

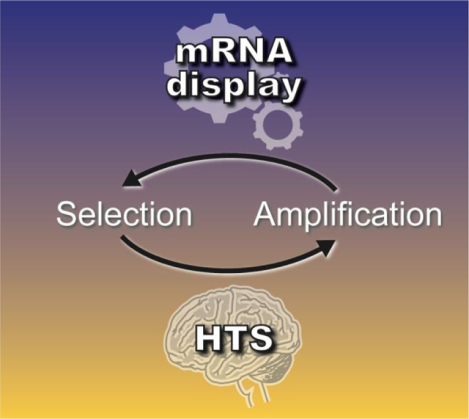

High-throughput sequencing (HTS) of mRNA display selection of functional peptides

## 1. Introduction

**Figure 1.**
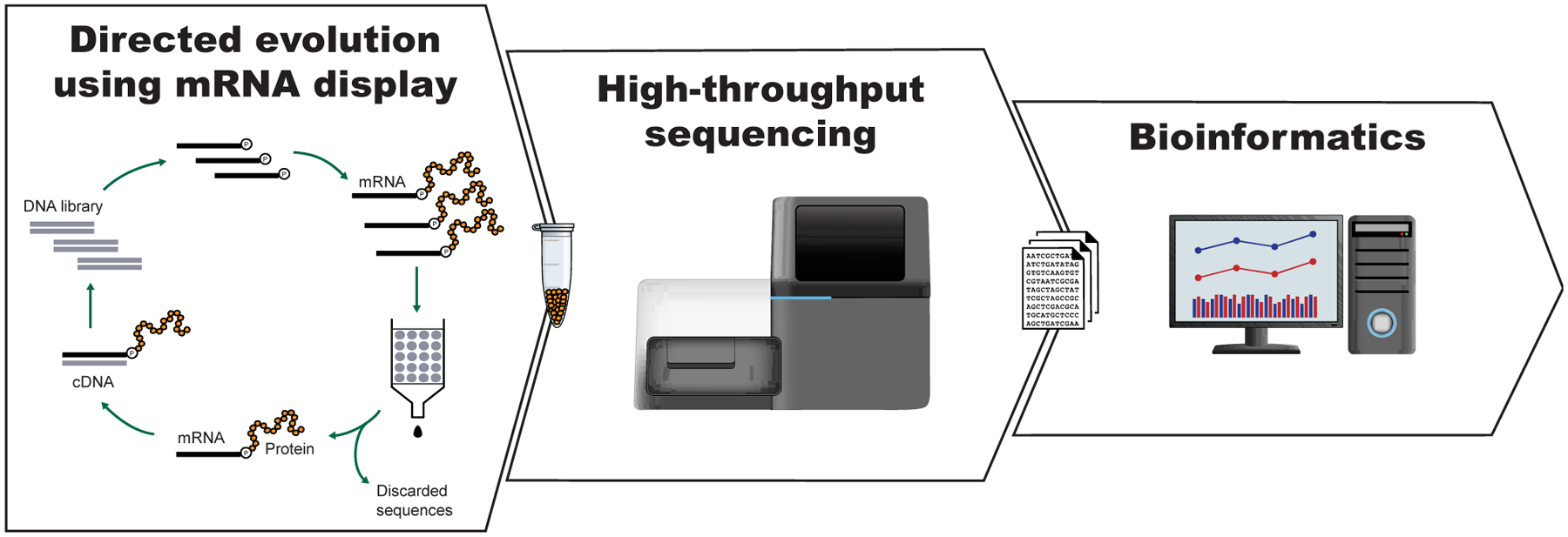
General scheme for the isolation of active sequences from *in vitro* evolution experiments, HTS and the analysis of the sequencing data. A large library of mutant variants is subjected to a selection process in which survival depends on the ability to carry out a specific biochemical function (e.g., binding). Selected variants are isolated and amplified while unselected variants are discarded. The cycle of selection and amplification is repeated several times (rounds) until variants with high activity dominate the library. The final library (and possibly intermediate pools) is then sequenced, such as by using high throughput sequencing (HTS) technologies. Finally, HTS data is analyzed using bioinformatic tools appropriate for the project’s goal. For a more detailed explanation of the selection process see Figure 2.

The development of peptides and proteins with desired activities is one of the great challenges of biotechnology. While rational design is an active field of research and can be applied in certain cases^1–4^, very often, the sequence-structure-function relationships are not understood with sufficient depth to employ rational design. In these cases, ‘irrational’ evolutionary strategies can be used, in which candidate sequences are selected or screened from a large number of variants on the basis of activity. Such methods have been highly successful, as recognized by the 2018 Nobel Prize in Chemistry. *In vitro* selection is of particular interest to physical biochemistry due to the ability to carefully control the selection environment, which enables probing of a variety of phenomena, including molecular interactions, folding behavior, and evolutionary pathways. During *in vitro* selection, functional molecules with desired properties are isolated from very large pools of random (or semi-random) sequences. This is achieved through iterative cycles of selection, amplification, and mutagenesis, until the final pool is sufficiently enriched with variants exhibiting the desired properties (Fig. 1). While it is often applied to nucleic acids, *in vitro* selection, when combined with peptide display technologies, can identify functional peptides of interest. In particular, mRNA display is widely used for this purpose, and it will be the focus of this Perspective.

High Throughput Sequencing (HTS) data analysis for *in vitro* evolution experiments has become increasingly common in the last decade for DNA and RNA molecules. However, this approach has not been as widely applied to mRNA display selections. The focus of this Perspective is to examine possible applications of mRNA display and HTS in contexts relevant for the field of physical chemistry, such as the improvement of binding kinetics measurement and the understanding of molecular fitness landscapes. We discuss the capability of HTS analysis applied to *in vitro* selections of peptides and review recent progress made in the field. Based on our experience, we discuss some possible issues that might arise during the sequencing process or the data pre-processing steps. Finally, we offer our perspective on possible future applications of HTS and mRNA display experiments, and discuss the potential effect that future improvements in sequencing technology might have on the field.

## 2. *In vitro* selection of displayed peptides

While directed evolution itself can be carried out at an organismal level, molecular selection techniques, which allow access to very large, diverse libraries, rely on linking phenotype and genotype. In other words, the physical isolation of a protein exhibiting a particular phenotype must also recover its sequence information, which can be amplified, mutated, and expressed. Thus display technologies are essential to the *in vitro* selection of peptides and protein ligands^5^. In this Perspective we focus on display techniques that accommodates pools of very high diversity, particularly mRNA display, and their combination with high-throughput sequencing (HTS) methods. In this section we introduce different display technologies and how mRNA display compares with them.

### 2.1. Phage display of peptides

Broadly speaking, protein display approaches can be classified into two groups: those that require the peptide libraries to be cloned and expressed using cells (cellular) and those in which expression and display are achieved without the need for cells (acellular). One of the most widely used techniques for functional peptide selection is phage display, a cellular technique in which the library proteins are displayed by fusion to an outer coat protein of a bacteriophage. The diversity of phage display libraries is fundamentally limited by the efficiency by which bacteria can be transformed, and is therefore up to ~10^9^ variants^5^. Typically, in this system, a short random sequence (encoding peptides roughly 6 to 45 amino acids long^5, 6^) is fused to a phage coat protein (e.g., the minor coat protein pIII, or, for short peptides, the major coat protein pVIII of the filamentous phage M13). A library of phages is created with each phage particle displaying the sequence encoded on its individual genome. The recombinant phages are produced and amplified using *E. coli*, purified, and subjected to selective pressure, such as binding to a molecule attached to a solid support. By cycling through the amplification and selection steps, phages displaying peptides with a desired phenotype can be enriched. The phage itself links the genotype (carried in its genome) with the phenotype (expressed as a fusion to its proteinaceous coat), and selected peptides can be sequenced after sufficient enrichment. Phage display is suitable for selection of peptides that can be used in antibody development, study of protein-protein interactions, and mediation of protein-ligand interactions^5, 7^. A limitation, however, is protein length, because peptides that are too long often interfere with phage assembly and/or infectivity, leading to an undesirable source of selection bias^8^. This can be somewhat mitigated by reducing the display valency, but other cellular approaches may be preferred when longer sequences are needed. Phage display has been extensively reviewed elsewhere^9, 10^.

The phage genotype-phenotype linkage is exploited in another cellular method, called Phage-Assisted Continuous Evolution (PACE)^11^. In PACE, a library of plasmids encoding an evolvable gene is transformed into *E. coli.* The host cells also contain plasmids that express phage proteins, but expression of an essential gene, pIII, is suppressed. Instead, pIII is only expressed if the selection plasmid has the desired activity, enabling production of a functional phage. Phage encoding enzymes with higher activity produce more pIII, in turn producing more viable phage that can infect more cells, propagating their genotype. The major advantage of this system is faster evolution allowing many generations of selection in tandem; as its name suggests, PACE is continuous and does not require manual intervention to cycle through a selection scheme. PACE has drawbacks, though, primarily in the difficulty of the experimental design and implementation, genetic engineering, and use of custom-built apparatus that may be difficult for a non-expert to implement.

### 2.2. Acellular display of peptides

A primary drawback of cellular approaches, whether phage display or otherwise, is library size, which is limited by the number of cells that can be infected or transformed by unique genotypes. In selection experiments, the probability of discovering sequences of high activity is proportional to the size of the initial library, all else being equal. In other words, the larger the initial library, the more likely one is to find the rare, high activity sequences, and thus larger libraries are fundamentally desirable. For standard proteins composed of 20 amino acids in which N positions are allowed to be variable, the size of sequence space is 20^N^ (≈10^1.3N^), which becomes experimentally intractable in the lab for N > 12 if one desires full coverage of the sequence space. Whether full coverage of sequence space is important depends on the scientific or engineering problem at hand. Full coverage of sequence space is of special interest for understanding fundamental questions about potential evolutionary pathways. However, if the purpose of the study is the engineering goal of identifying functional molecules, the completeness of coverage of sequence space is not intrinsically important. Instead, the frequency of sequences with the desired phenotype within the space of sequences explored, and its ratio to the library size, is the critical parameter, and is heavily dependent on library design. With sufficiently efficient selective amplification of active sequences, it should suffice to have a library large enough to have one (or a few) of the sequences exhibiting the desired phenotype. Large library sizes can be achieved by acellular methods, which are limited by physical constraints on concentrated biomolecular solutions (e.g., protein aggregation) rather than much smaller biological constraints such as the rate of transformation or infection.

Two widely used acellular approaches for protein selection and evolution are ribosome display and mRNA display (Table 1). Library diversity using these methods usually is 10^12^–10^14^ variants, surpassing the cellular methods by orders of magnitude^5, 12–14^. In addition to the increased library size due to lack of need for transformation, acellular approaches show reduced biases by avoiding cellular expression (e.g., the toxicity of protein sequences is not relevant in acellular approaches). Like cellular methods, acellular methods are amenable to combination with diversity-generating techniques that mimic natural evolution: error-prone PCR is used to introduce random mutations, gene shuffling (recombination) is used to generate permutations of mutations, and non-natural amino acids can be introduced.

In order to create the physical link between genotype and phenotype, both ribosome display and mRNA display take advantage of the fact that an mRNA and its encoded protein, while not covalently bound, are in intimate proximity during translation. Thus, manipulation of events surrounding termination of *in vitro* translation can capture an mRNA together with its newly expressed protein molecule. In ribosome display^15–17^, the mRNA and the translated peptide or protein product are held together non-covalently by the ribosome. To accomplish this, the stop codon of the gene is deleted, and therefore the ribosome does not dissociate at the end of mRNA translation. This ternary complex of mRNA, ribosome and peptide is further stabilized mainly through high Mg^2+^-concentrations and incubation at low temperature. While this complex can be stable for days, any subsequent selection conditions are limited to those that preserve the integrity of the mRNA-ribosome-peptide complex. When more physiological and/or stringent conditions are of interest, mRNA display is an important alternative (Table 1).

## 3. mRNA display

**Figure 2.**
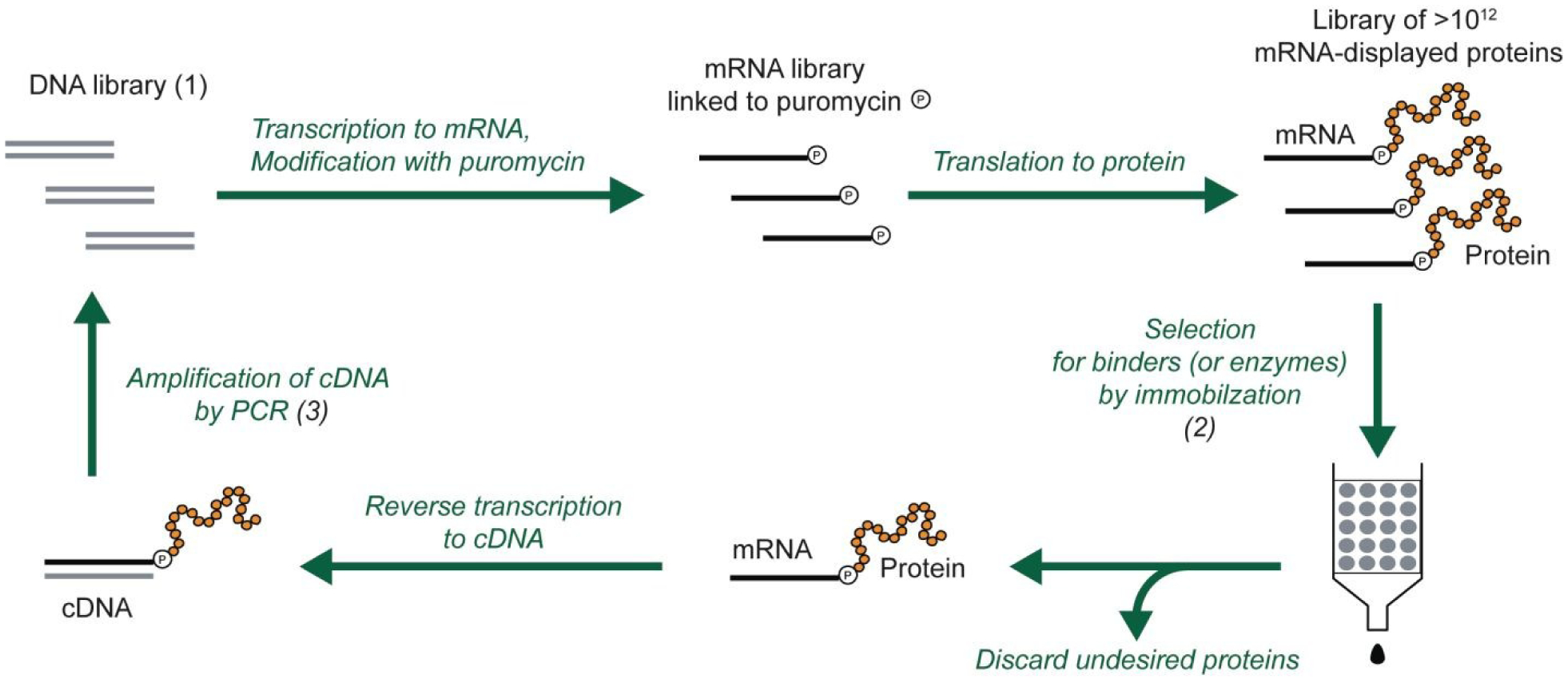
Selection and evolution of proteins by mRNA display. The procedure begins with a library of DNA (1) that encodes the library of protein variants. The DNA is transcribed into RNA, modified with puromycin and translated to mRNA-displayed proteins. In the selection step (2), the protein variants with the desired properties are separated from the undesired proteins. The selected variants are reverse transcribed to cDNA (can also be done before the selection step), and multiplied by PCR amplification (3). This round of selection and amplification is repeated until the resulting library is dominated by proteins with the desired properties. For protein evolution, the amplification step can be modified to introduce additional diversity (e.g., mutations).

mRNA display is a selection and evolution technique for functional peptides and proteins that is performed entirely *in vitro*^18, 19^. Like ribosome display, mRNA display can interrogate very large libraries of peptide variants, being orders of magnitude larger than libraries of other display technologies^20^. However, instead of using a ribosome to connect the mRNA and its peptide in a non-covalent ternary complex, the mRNA itself is covalently attached to the peptide. In an idealized sense, mRNA display is a minimalistic version of display techniques as the genotype (mRNA) and the phenotype (polypeptide) are connected by covalent linkage through a small molecule. Specifically, the 3′-terminus of the mRNA is modified by the small molecule antibiotic puromycin, which is a structural mimic of a charged tRNA and bears a free amine analogous to that of an aminoacyl-tRNA (Fig. 2). At the end of *in vitro* translation of the mRNA into the corresponding protein, the puromycin is ‘mistaken’ by the ribosome as a charged tRNA and then covalently linked to the nascent peptide chain. After this reaction, the ribosome can be dissociated and the mRNA-displayed protein can be isolated and used as desired. This stable covalent link in effect renders every RNA sequence encoding its polypeptide directly selectable by the polypeptide’s phenotype, and also amplifiable after selection by reverse transcription and PCR.

Progress of the selection is commonly monitored by measuring the recovery of mRNA-displayed proteins during the selection step, which is expected to increase over rounds if active variants are being selected. This measurement estimates the bulk activity (binding or catalysis) of the library of enriched variants. When the desired variants have been sufficiently enriched, the proteins are identified by DNA sequencing and subsequently analyzed individually as appropriate for the particular activity. In a complementary approach, the progress of the enrichment can also be monitored through DNA sequencing of the library after each round of selection. The comparison of populations of variants over the course of selection can reveal the enrichment of dominant proteins.

As with other selection techniques, the general biophysical nature of the selectable entity should be kept in mind. In this case, the mRNA-protein fusion is mostly RNA by mass. With an RNA monomer being roughly three times the mass of an amino acid and each codon being three nucleotides long, the mRNA-protein fusion is approximately 1/10 protein by mass. A fortunate consequence of this is that the fusion benefits from the high solubility of the negatively charged RNA. Thus, while random protein sequences are prone to aggregations^21^, random mRNA-protein fusions are less so. Solubility can be further improved by a selection preceding the intended selection (a ‘pre-selection’), in which the soluble fraction itself is selected. On the other hand, the covalent linkage of the mRNA to the peptide means that it is possible for an mRNA-protein fusion to survive a selection due to activity of the mRNA, not the peptide. For example, a selection may inadvertently result in ribozyme or aptamer sequences. This outcome can be guarded against by strategies such as ‘protection’ of the mRNA in a duplex with complementary DNA. It is also possible that the presence of the RNA affects the peptide’s activity, e.g., by effects on folding, such that a fusion exhibiting a particular activity may not exhibit the same activity when expressed as an isolated peptide. In addition, the selection step often requires experimental measures that should be kept in mind during data interpretation, such as the need to attach an affinity tag to the substrate to render selected molecules isolable. As with any *in vitro* selection experiment, the resulting ‘hits’ must be validated by additional assays. Nevertheless, due to the minimalist display design, the stability of the covalent link, and the freedom to operate under a wide range of conditions in this *in vitro* format, mRNA display is a powerful method for peptide or protein display systems.

mRNA-displayed peptide and protein libraries have regularly been selected to isolate protein binders and, in some cases, even enzymes^22, 23^. For example, mRNA display has been utilized to study protein-protein interactions, or interactions between proteins and small molecules or other targets^24–26^. mRNA display has also been used to display cyclic peptide libraries, enabling the discovery of bioactive macrocycles as potential drug candidates^27–29^. More detailed reviews of the mRNA display technology and its applications can be found elsewhere^24, 28, 30–33^. Furthermore, mRNA display has proven to be particularly suitable for the investigation of fundamental questions. For example, mRNA display selection can be used to discover entirely *de novo* proteins from libraries of randomized polypeptides, with implications for the potential origin of the earliest functional proteins^34, 35^. Another example is using mRNA display to mimic natural Darwinian protein evolution in the lab to examine protein fitness landscapes^36–39^. The high versatility of mRNA display methods adds value to its potential benefits for the *in vitro* selection of peptides and proteins.

## 4. High throughput sequencing of mRNA display selections

Sanger sequencing has been widely used to analyze the outcome of mRNA display selections. While the low throughput of Sanger sequencing is usually sufficient to identify the winning proteins from a highly enriched library at the end of a selection, additional information on alternative variants with lower abundance is limited. Also, the abundance of a sequence often does not accurately predict its activity, and even enrichment can be a noisy correlate to binding affinity^38, 40^. Monitoring the progress of enrichment throughout a selection by Sanger sequencing is also very challenging or even impossible because the active protein variants are in the vast minority during all but the last rounds of selection. In contrast, high throughput DNA sequencing can overcome these challenges. A sequencing depth of millions of reads potentially allows for the identification of many more active proteins with a wider range of activities, as opposed to only the most abundant variants.

High-throughput sequencing (HTS) refers to a number of technologies capable of producing a large amount of sequence data (Table 2). HTS methods are highly scalable, with some allowing a large number of different variants (thousands to millions or even billions) to be sequenced in parallel. HTS methods are also referred to as next-generation sequencing (NGS) or second-generation sequencing (2GS) in the literature. However, the terms NGS intuitively refers to the most recent sequencing technology, hence, it has been progressively abandoned in literature since the advent of more recent long-read sequencing methods. In the last 20 years, the data output capacity has outpaced Moore’s law and the associated costs have dropped almost at the same rate. While the sequencing of one entire human genome in the Human Genome Project took 13 years and cost nearly three billion dollars^41^, nowadays many whole human genomes can be sequenced within a single day for approximately a thousand dollars each. HTS technologies have tremendously impacted several fields of biological research and have opened the door to new approaches in medicine, such as in personalized medicine^42–44^.

The sequencing market is nowadays dominated by the Illumina platform. However, several other companies offer sequencing platforms that use different technologies (Table 2), which present different advantages and disadvantages. Illumina’s high popularity is mainly due to the sheer throughput and low cost, such that for genomic sequencing applications high coverage can be readily obtained. However, a major limitation is the length of individual sequence reads. For longer reads (more than a few hundred base pairs), other sequencing technologies are preferable or necessary (Table 3). Sequencing platforms capable of long reads usually have a higher error rate associated with them, but often strategies can be devised to circumvent this problem (e.g., multiple effective reads of the same base), and technologies are constantly under development in this highly competitive area. Detailed comparisons among different sequencing methods can be found elsewhere^45–48^.

In mRNA display selections, HTS enables the tracking of evolutionary paths of selected sequences throughout the selection and evolution process, as well as measurement of the distribution of activity of proteins over sequence space. A clear impact of HTS is that the high depth of sequencing can reveal a greater number of active sequences, especially those without closely related neighbors. Although such sequences might be rare, they could exhibit high activity and thus be of interest. Also, a practical benefit of deep sequencing is the potential for reducing the number of cycles required to identify active clones. Without HTS, a selection is usually pursued until the active variants represent a majority of the library, so that a small number of clones subjected to Sanger sequencing would identify the ‘winning’ sequences. With HTS, however, the selection can be stopped relatively early and clones selected on the basis of the rate of their enrichment, even if they are present at somewhat low relative abundance (e.g., <1%)^40^. In summary, large sequencing depth while tracking selections can enable both improved identification of active clones as well as a better understanding of the evolutionary process. We discuss in Section 5 below some important applications in which NGS is transforming our understanding of fundamental problems.

Given the unprecedented increase in data, an interesting question is whether NGS can allow one to entirely circumvent the evolutionary process during discovery of functional peptides from a diverse library. With unlimited sequencing capability, one could imagine sequencing the starting library, subjecting the library to a single screening reaction, and then sequencing the selected pool. In principle, a comparison of the composition of the pool before and after the screen should yield estimates of the relative activities of all of the different sequences in the library. Whether this is attainable in practice depends on the library size. In directed evolution experiments, initial libraries are generated to, ideally, cover as much sequence and structural diversity as possible while targeting the activity of interest; the larger the library, the greater the chance of discovering rare, active sequences. Libraries generated using mRNA display methods can typically contain up to ~ 10^14^ different variants. While Sanger sequencing could yield perhaps a few hundred sequences, massively parallel HTS methods can read up to 10^10^ (as of 2019, Illumina’s NovaSeq 6000 System can yield a maximum of 20 billion reads per run^49^). However, despite the high number of different variants that can be sequenced nowadays, the number of variants that can be explored experimentally in the initial library is still higher. Therefore, if the initial library has fewer than ~1 billion variants, it is conceivable to use HTS effectively as a screen. But if mRNA display is used for exploring extremely diverse libraries, at least a few rounds of selection are currently necessary to reduce the complexity of an mRNA display library to a tractable size.

## 5. Molecular fitness landscapes

In molecular evolution, the fitness of a sequence is a quantitative measure of its evolutionary favorability, and can be defined in multiple ways *in vitro*, depending on experimental context. The function of fitness in the multidimensional space of all possible sequences is known as the fitness landscape. In simplified terms, the fitness landscape can be described as a series of peaks (corresponding to families of related sequences with elevated fitness) emerging above the background (corresponding to regions in the sequence space of low or zero fitness). Evolution over the fitness landscape can be conceptualized as a random walk with a bias toward climbing hills^37, 50^. Knowledge of the fitness landscape is critical for understanding molecular evolution, as evolutionary outcomes could be predicted, in principle, given complete knowledge of the fitness landscape in a particular environment. However, prior to HTS, empirical data on fitness landscapes was quite limited.

To understand the importance of HTS for examining fitness landscapes, let us consider how the shape of these landscapes influences natural selection. Under conditions in which selection pressures are strong, as is common during *in vitro* selection, sequences evolve by local uphill climbs over the landscape. Over a perfectly smooth peak, one could imagine easily reaching the global optimum through a continuously uphill climb. However, if the landscape contains many local optima with valleys separating them from the global optimum, populations of sequences may become trapped on the local optima^37, 51^. Thus, the ability of natural selection to discover an optimal sequence is heavily influenced by the ruggedness of the fitness landscape.

One measure of ruggedness is epistasis, which describes how different sites along the sequence interact to determine the fitness contribution of each mutation. In other words, in a landscape with epistasis, the genetic background of a mutation influences how beneficial or detrimental that mutation is. Sign epistasis describes the situation in which the effect on fitness of a single mutation is either positive or negative depending on the presence or absence of another mutation. Reciprocal sign epistasis corresponds to a particular case of sign epistasis in which mutations that are independently advantageous became jointly unfavorable (or vice versa). Such epistasis is particularly important for the landscape, as it leads to local optima^52^. Epistasis is therefore an important feature determining the viability of individual evolutionary pathways of protein sequences^37^. Calculations to measure epistasis in experimental fitness landscapes have been reviewed elsewhere^53^.

**Figure 3.**
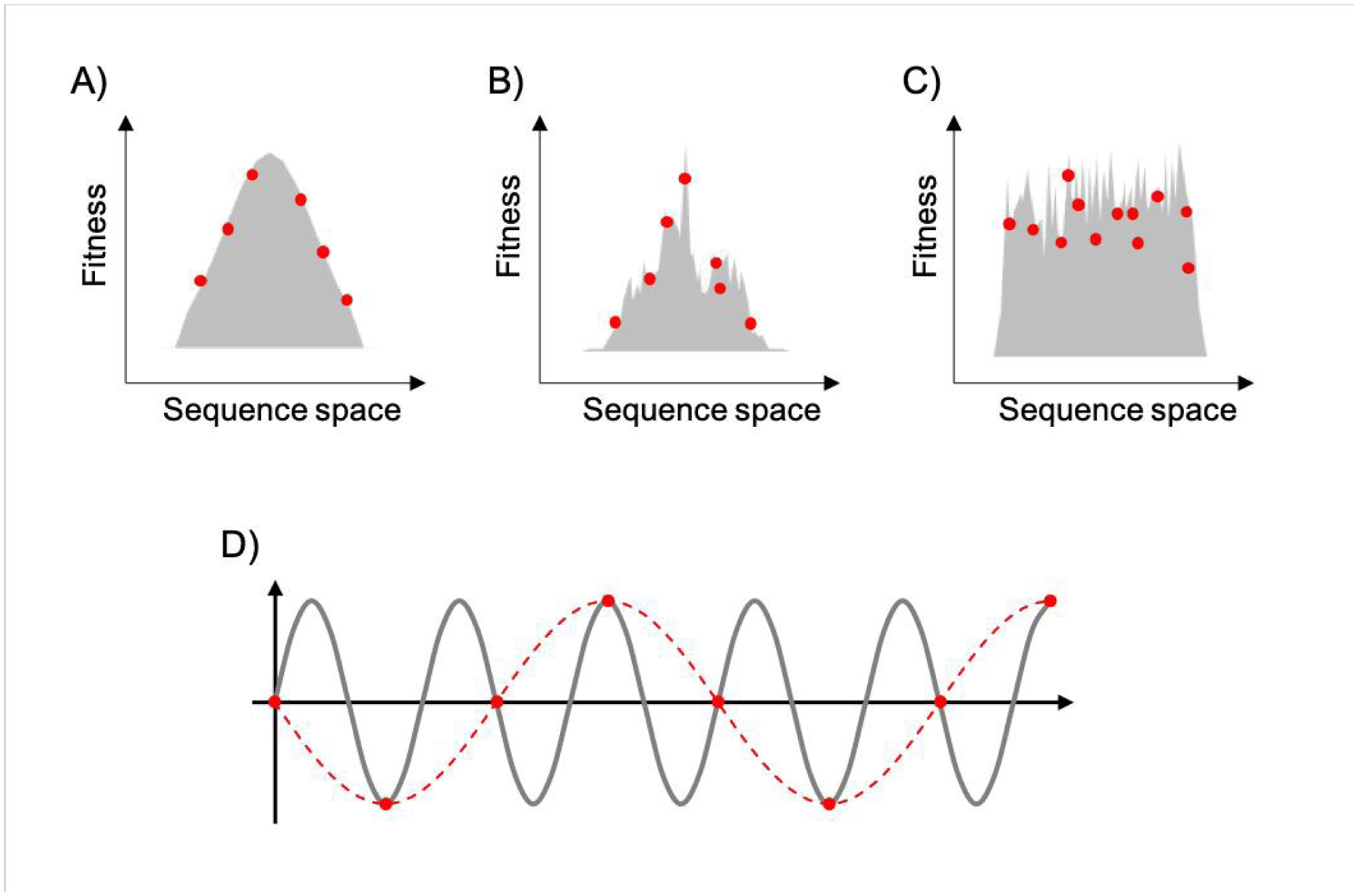
Representation of fitness landscapes with different levels of ruggedness. Simplified 2D visualization of A) a smooth landscape (Mount Fuji type), B) an intermediate ruggedness landscape, and C) a highly rugged landscape. Red dots correspond to random sparse sampling on sequence space. In D), the gray line corresponds to a sine wave of frequency *f*, red dots correspond to sparse sampling below the critical sampling frequency (f_S_<2f), and red dashed line corresponds to the aliased wave reconstructed from undersampling.

To map fitness landscapes, individual sequences would be sampled and their fitness determined (such as by sequencing). Let us consider how the depth of sampling influences our ability to probe ruggedness and epistasis. In the most simple, smoothest landscape (i.e., that with a single peak), the absence of local maxima implies that under a regime of strong selection and weak mutation, evolution starting from any point in sequence space will end in the global optimum. Generally, sparse random sampling on these topographies will still give an adequate representation of the landscape, because the fitness of unsampled points in sequence space can be interpolated using an assumption of additivity among mutations (Fig. 3a). In contrast, a highly rugged landscape would occur if the fitness of related sequences were totally uncorrelated (Fig. 3c). Evolution on this type of landscape will almost certainly not end in the global optimum, and populations will be ‘stuck’ in peaks corresponding to local maxima. In these topographies, sparse random sampling cannot give a proper representation of the landscape. Although these two cases (completely correlated and completely random fitness landscapes) are interesting as theoretical limits, most empirical landscapes exhibit an intermediate degree of ruggedness (as well as a certain degree of correlation), lying somewhere in between these two limiting cases. (Fig. 3b). The ruggedness of the landscape ultimately determines whether subsampling of sequence space can result in a trustworthy representation of the topography. Severe undersampling of a rugged landscape would miss many epistatic correlations. For realistically rugged landscapes, high sampling levels, enabled by HTS, are essential for understanding the fitness landscapes.

The issue of adequately sampling fitness landscapes resembles the core idea behind the Nyquist–Shannon sampling theorem for digital signal processing^54^. In this field, sampling refers to the process of converting a continuous signal into a string of discrete values. The theorem states that, for a given continuous function, there is a critical, minimum rate of sampling for which perfect reconstruction of the function is guaranteed, this rate being at least twice the maximum frequency response of the signal. That is, f_N_=f_S_/2, where f_N_ is the critical frequency (also called Nyquist frequency) and f_S_ is the sampling frequency. Similarly, for a given sample rate, there is a maximum bandlimit or frequency that ensures perfect reconstruction. For example, in the case of a sine wave, sampling at less than twice the maximum frequency will lead to a lower frequency sine wave. This phenomenon is known as *aliasing* (Fig. 3d). Sampling at more than twice the maximum frequency ensures perfect reconstruction of the wave function. In the case of fitness landscapes, whether the number of sampled sequences is large enough to reconstruct the topography of the fitness landscape depends on the topographical features of the landscape, which is determined by the epistatic interactions. In this context, rugged landscapes are at a higher risk of suffering ‘aliasing’.

In addition to mapping epistatic landscapes, which is discussed further in Section 6, the combined use of mRNA display libraries and HTS methods can provide a direct view into the evolutionary history of peptides over the course of selection^37^. Like Sanger sequencing, HTS can identify the different families of sequences selected for high activity at the end of the selection, but with higher depth. Additionally, it can provide valuable information on sequence composition at different points of the selection, i.e., ‘snapshots’ during evolution. At each snapshot, deep sequencing can reveal the number of families of similar sequences, the size of each family present, as well as information about the common motifs of a family or the different motifs across families. Merging the sequencing data across rounds of selection thus can provide a window into the details of the evolutionary process. For example, one can estimate how the number of families and their sizes changed over the selection, at which point of the selection the different families emerged (or were left behind) and how the different families compete with and related to each other. Importantly, one may potentially trace the evolutionary trajectory of the most active families across different rounds of selection.

## 6. Recent applications combining mRNA display and HTS

The analysis of High Throughput Sequencing (HTS) data from *in vitro* evolution experiments has only recently been applied to mRNA display selections. So far, studies combining both techniques have primarily focused on understanding the effect of epistatic interactions in a peptide fitness landscape, improving the characterization of peptide ligands and mapping a protein-protein interactome. These promising studies highlight the potential benefits of using HTS to understand and predict evolutionary pathways, and to accelerate the quantification of peptides’ binding affinities.

### 6.1. Epistasis

Many empirical examples of epistasis are known in local sequence space for proteins, in which a combination of mutations has an effect deviating from the sum of the effects of the individual mutations, but systematic characterization of epistatic interactions through larger sequence spaces was challenging, and indeed a herculean task, before HTS. In 2014, Olson and colleagues quantified the effects of all pairwise epistatic interactions in the IgG-binding domain of protein G (GB1, 56 amino acids in total) using a combination of mRNA display and HTS^39^. The relative binding ability of all single and nearly all double amino acid mutants of IgG-FC was estimated by measuring the frequency of each variant before and after affinity enrichment. Negative epistasis (in which fitness of the double mutant is decreased compared to the linearly added effects of the single mutants) was found to be dominant and to occur between combinations of destabilizing mutations, i.e., combining two deleterious mutations gave a double mutant that was even worse than expected. The predominance of negative epistasis had been previously observed in protein enzymes and RNA molecules, suggesting that negative epistasis may be a common feature of biological parts^55–58^. In comparison, positive epistasis (in which fitness of the double mutant is increased compared to the linearly added effects of the single mutants) was found to be rare. These quantitative comparisons, requiring measurement of hundreds (or more) of mutants, are essentially enabled by HTS, as collecting the requisite data would be extremely tedious by other means.

Another important finding was that many mutations that were generally deleterious were found to be beneficial in at least one alternative mutational background. This is relevant because, although rare, positive epistasis can substantially expand the functional portion of sequence space, and thus, the accessible evolutionary pathways. Again, the depth of data from HTS was required to discover these rare situations, which may have an outsized impact. An illustrative example of the importance of these rare pathways came in 2016, when the same group used mRNA display and HTS to experimentally characterize the fitness landscape of four amino acid sites in protein GB1, corresponding to 20^4^ = 160,000 variants^36^, including several mutations with interactions known to be positively epistatic^39^. Reciprocal sign epistasis (i.e. mutations that are separately advantageous became jointly unfavorable) blocked many direct evolutionary paths through genotype space^59^, leading to an appearance of difficult optimization over the local landscape. However, these ‘dead end paths’ could be circumvented by following longer indirect paths through consecutive gains and losses of mutations. In other words, they are overcome through reversible mutations that avoid the need to lose fitness at any particular step. This mechanism allows protein optimization by natural selection (i.e., uphill climbs) despite epistasis. The indirect paths reduce the constraint on adaptive protein evolution, supporting the idea that the previously ignored regions of the functional sequence space may be crucial for the evolution of proteins. This highlights the qualitative importance of HTS, which allows much deeper exploration of sequence space and discovery of rare but important features, for understanding evolutionary trajectories.

### 6.2. Accelerated discovery and characterization of peptide ligands

A major application of *in vitro* selection techniques is the generation of high affinity polypeptide ligands against individual targets of interest. Usually, several rounds of selection are performed until most clones are functional (enriched)^60^. Recently, a new approach combining mRNA display with continuous-flow magnetic separation analyzed by HTS has markedly accelerated the process, to the point of achieving the selection of human IgG binders with nanomolar affinities in only a single round^61^. This highlights the practical benefit of HTS in saving experimental time and resources.

One consequence of using HTS to analyze *in vitro* selection is that one may obtain quite a long list of candidate sequences. Additional experiments are required to quantify relative binding affinities for each candidate sequence. Even for a relatively small number of sequences, this characterization step is often labor-intensive and represents an experimental bottleneck in analysis of selected sequences. Given results from HTS, the problem is seriously compounded by the number of candidates. To solve this problem, the Roberts group recently used a combination of mRNA display and HTS to calculate the on- and off- rates for many thousands of mRNA-displayed ligands simultaneously, without synthesizing or purifying individual sequences^38^. To do so, they devised a method based on the fact that the on- and off- rates of a sequence (*k*_on_ and *k*_off_) determine its fractional presence at different time points during the selection step. That is, sequences with high on-rates are present in higher fractions at early time points because they bind quickly to the target; however, at later time points, as the fraction of ligands with slower on-rates bound to the target increases, the fraction of the ‘fast’ ligands bound to the target decreases. Following this idea, they mixed a library of mRNA–peptide fusions with an immobilized target and removed an aliquot at different time points for washing, PCR, and deep sequencing. The resulting HTS data yielded the identity of all the ligands bound to the target at each time point and their frequencies, which could be used to calculate the on-rates of each ligand. Off-rates could be measured in an analogous fashion, and binding affinities (*K*_d_ = *k*_off_/*k*_on_) were therefore obtained for thousands of ligands in parallel. This example illustrates the creative use of HTS for not only tracking sequences during evolution, but also for massively parallelizing a binding assay.

### 6.3. Interactome

An interesting application of HTS in mRNA display capitalized on the fact that its depth enables analytical coverage of the proteome, allowing production of high-throughput protein-protein interactome datasets^25^. In 2012, Fujimori et al. described the first complete interactome for proteins that interacted with mouse interferon regulatory factor 7 (Irf7), by using mRNA display technology combined with HTS^62^. The accuracy of the analysis was validated by comparing the results with real-time PCR assays for randomly selected interacting regions. The high degree of overlap between the positives found from the HTS analysis and those from the real-time PCR assays confirmed the high reliability and coverage of the method. An advantage of this method over others (e.g., yeast two-hybrid system) is the ability of mRNA display to access a larger sequence space of potential proteins.

## 7. Possible issues and suggested practices

The high depth of HTS analysis requires additional attention to detail and can reveal biases in the selection, the library design or the library synthesis that would otherwise be undetectable or considered minor. In addition, choices during the sequencing and data analysis can strongly influence the error rate and the number of full-length sequences recovered at the end of the bioinformatic pre-processing. Some aspects of the sequencing and preliminary bioinformatic treatment that may be considered to produce acceptable processed data and facilitate posterior analysis are discussed here, based on our experience with Illumina data.

### 7.1. Library design

The presence of conserved regions in the library can be important for experimental reasons, such as preserving a structural scaffold. Conserved regions are also useful for alignment during bioinformatic analyses. However, these regions can be problematic for HTS^63^. Low-diversity amplicon libraries have documented quality score issues^64^. For example, in the Illumina sequencing-by-synthesis platform, the location of PCR colonies generated by individual template molecules is determined by finding fluorescent spots on an image during the early cycles of synthesis. If there is a high density of spots of the same color, as would occur when a large fraction of templates have an identical constant region, discrete spots cannot be reliably identified, leading to low quality. Thus, some level of nucleotide diversity at each position is important for the generation of high-quality data. Two possible causes of low diversity are library design and the convergence of the selection process. First, even highly randomized libraries usually contain conserved regions on the 5’ and 3’ ends for PCR primer complementarity, inducing low diversity at the beginning of the sequencing run. Second, after a successful selection process, later rounds are typically dominated by a few families, resulting in low nucleotide diversity.

There are a few means to overcome these issues. One method to combat the problem of overlapping fluorescent spots is to reduce the density of spots by diluting the sample, thus sacrificing sequence depth for higher quality. Other methods include increasing nucleotide diversity^65, 66^. For example, one may add (‘spike in’) a sample of high diversity such as the ΦX174 genome. This genome is from a small, well-characterized bacteriophage that has a relatively uniform base composition (and was incidentally the first whole genome to be sequenced). Sequencing reads derived from this genome can be readily removed during bioinformatic processing. Spiking in ΦX174 DNA increases the sample diversity at the beginning of the read, improving intensity distribution issues during initial reading of the template. Depending on the specifics, it might be necessary to spike in between 5% and 50% ΦX174 DNA to achieve a sufficiently diverse sample^67^. As with the method of sample dilution, the main disadvantage of spiking a high amount of ΦX174 is the loss of sequencing depth for the desired sample. Ideally, the amount of ΦX174 used should achieve a good balance between improvement in sequencing quality and loss of reads.

An alternative method to increase nucleotide diversity without sacrificing sequencing depth is the use of degenerate insertions after the adapter constant region. To increase the diversity of the pool, a series of random nucleotides can be added to the adaptor region, after the primer binding site^68^. If the added series of random nucleotides is of varying length (e.g. 2, 4, and 6 nt), this addition can improve sequence diversity by essentially frame-shifting the sequences with respect to one another. This increases the diversity in not only the initial primer but also beyond it, due to the frame shift, and is likely superior to spike-in or dilution methods. However, while the spike-in and dilution methods can be applied to samples that have already been prepared (i.e., after an issue has been identified), the addition of a small randomized region would require design of additional PCRs and fresh sample preparation. Since these methods are not mutually exclusive, a combination of methods could be considered for particularly problematic cases.

An interesting advantage of HTS is the identification of minor anomalies in the constant regions that may have been otherwise overlooked during selection. These unanticipated insertions, deletions and substitutions might be either functional (i.e., selected) or non-functional (e.g., primer synthesis errors or sequencing errors). Knowledge of expected error rates and profiles during both synthesis and sequencing can be helpful, and the overall error rate from each run should be compared to standard error rates obtained using that technology. In general, we find that it is most useful to have a method for independent reads of the same template (e.g., in Illumina sequencing, paired-end reads with as much overlap as possible; or in PacBio sequencing, consensus sequencing) in order to reduce the error rate. In any case, anomalies point toward a need for further consideration.

### 7.2. Library synthesis

Library synthesis is a critical step in mRNA display selections. In principle, an optimal library can be chemically synthesized using a trimer-block system to control codon type and frequency, and to prevent the introduction of premature stop codons. While trimer-block synthesis is the ideal method of library generation, slight impurities can undermine selections and downstream analyses and therefore should be kept in mind. For example, synthesis can be contaminated by monomer, dimer, or tetramer blocks, which introduce frame shifts that can result in undesired codons and truncated sequences. While these sequences might be lost during selection, preliminary data from our mRNA display studies suggests they persist and may be amplified in at least some conditions. Therefore, depending on the application, it may be critical that libraries are purified (e.g., size-selection by HPLC), that quality control data is obtained from the supplier, and that researchers independently confirm library purity by HPLC, capillary electrophoresis, high-resolution gel electrophoresis, or preliminary sequencing.

### 7.3. Sequencing

In single-end reading, the template is read in one direction (from one end to the other), while in paired-end reading, the template is read from both directions, resulting in forward and reverse reads of the same template sequence. Single-end reads are more economical, but pair-end reading offers increased quality with low error rates and also helps identify insertion and deletion errors in sequencing, which cannot be distinguished from true insertions and deletions in the DNA during single-end reading. Double coverage by paired-end reading allows creation of a consensus sequence having low error rate (e.g., given 1% error per base in one direction, paired-end error rates can be 0.01% if all sequences containing disagreements are discarded)^69, 70^, reducing the chance that errors introduced from sequencing are carried over into downstream bioinformatic analyses.

### 7.4. Bioinformatic pre-processing of data

**Figure 4.**
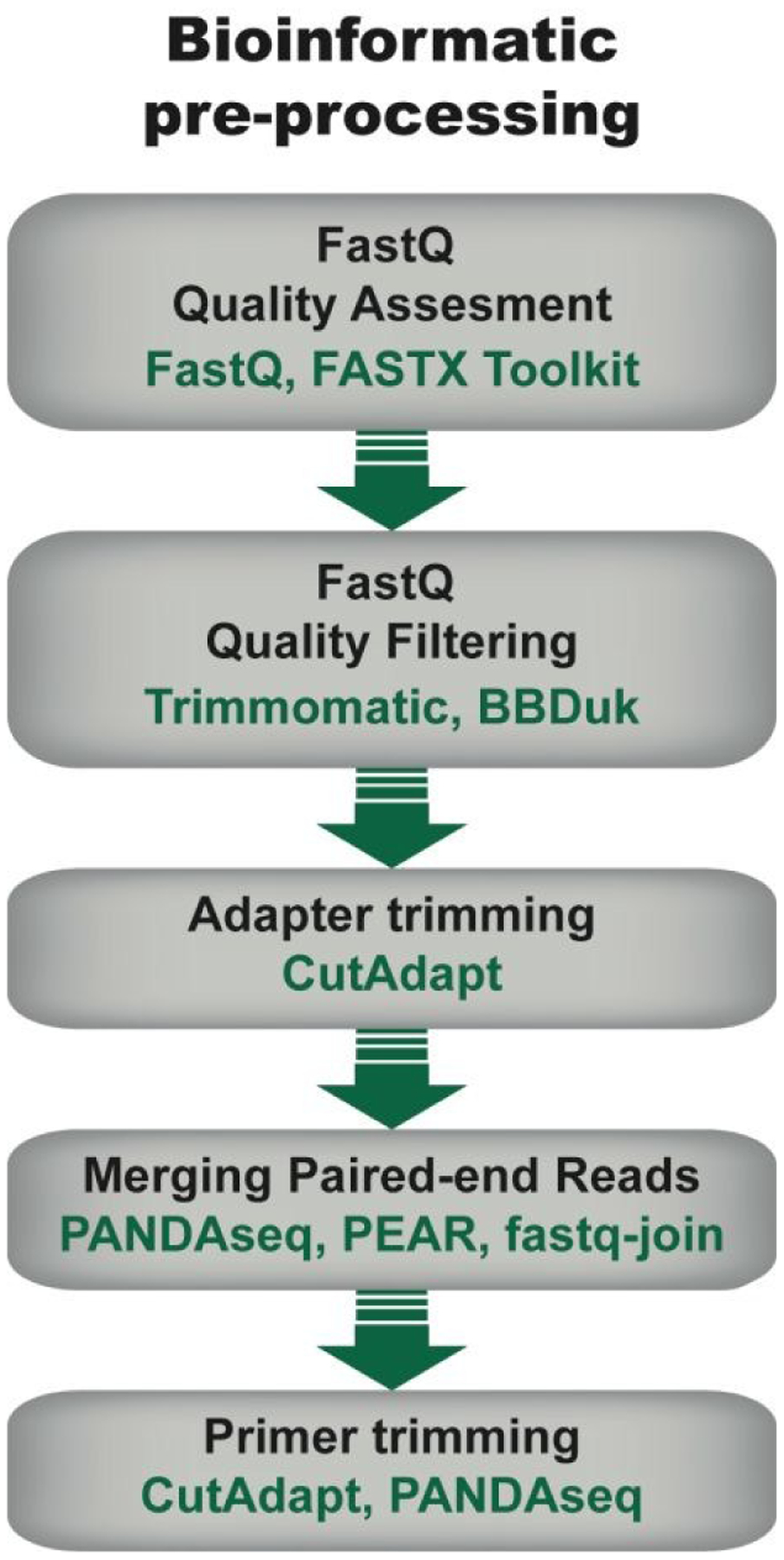
General flow chart for bioinformatic pre-processing of high throughput sequencing data for mRNA display. Pre-processing steps include an initial quality assessment of the fastq sequences (i.e., sequencing data with associated quality metrics), quality filtering to discard sequences of low quality, adapter trimming in the case of amplicons shorter than the read length, joining of pair-end reads, and primer trimming. Some examples of tools that can be used at each step are given. Note that the use of each step depends on the data and desired downstream analysis.

Pre-processing of sequencing data is imperative for successful downstream data transformations and analyses (see refs.^71–73^ for some examples and discussion). Pre-processing steps typically entail an initial quality assessment, removal of low-quality reads, quality trimming, adapter trimming, read joining/merging for paired-end reads, and primer trimming/sequence extraction (Fig. 4).

#### Quality Assessment and Filtering:

The initial quality assessment gives a look into read length and quality distributions to check conformity to expectations. This step can be implemented using tools like FastQC^74^ or FASTX Toolkit^75^, among others. Ideally, reads will have near-uniform length, matching the expected length based on amplicon size and sequencing method, and high average quality scores. In a standard, high-quality sequencing run, >95% of reads will have an average read quality score >Q30^76^. Unusually low quality scores can be a criterion for removing low quality reads, if desired. Under ideal conditions, Q30 is equivalent to the probability of an incorrect base call being 1 in 1000 times, which corresponds to a base call accuracy (i.e., the probability of a correct base call) of 99.9%. While Q30 is considered a benchmark for quality in HTS, quality scores are only based on instrument metrics, thus, they are usually higher than the true quality. Spiking in a standard sequence such as ΦX174 DNA and evaluating its error profile may give a more accurate estimation of sequencing error^77^. For a comprehensive review on error correction and available tools, see ref.^78^.

During quality assessment, it is important to consider the library’s properties and sequencing method, and how they will influence quality scores. For example, low-diversity libraries and read lengths >150bp will typically have lower average quality scores than high-diversity libraries sequenced with short read lengths. In addition, it is expected in Illumina sequencing that quality decays later into the read. Therefore, reads can be quality-trimmed to remove low-quality bases with tools such as Trimmomatic^79^ or BBDuk^80^, using parameters informed by the quality assessment (e.g. distribution of low-quality bases and their scores). This step ensures that only high quality bases are retained, which enables optimal read joining (for paired-end sequences) and reduces error-based noise in downstream analyses.

#### Adapter trimming:

In cases where the amplicon being sequenced is shorter than the read length, adapter sequences will be found on the 3’ end of the read, so an adapter trimming step might need to be implemented, using tools similar to Cutadapt^81^.

#### Merging paired-end reads:

Next, in paired-end sequencing, the full amplicon is reconstructed by joining (or merging) forward and reverse reads using tools such as PANDAseq^82^, PEAR^83^, or fastq-join^84^. Depending on the desired application, the joining process should be optimized to allow the maximum overlap between forward and reverse reads, while minimizing mismatch allowance. Maximum overlap will ensure that the regenerated amplicon is of the highest possible quality (in general, for each base pair in the overlap, the higher quality base is retained) and minimize the chance of introducing a frameshift. Mismatch allowances should be determined based on overlap length and quality of bases in the overlapping regions. The probability of reading a frame shift or ‘mutation’ from sequencing error will increase with the number of mismatches allowed (i.e. more mismatches allow more frameshifts and mutations). Conversely, the number of reads that are joinable will decrease as the number of mismatches allowed decreases (i.e. more stringent tolerances allow fewer joined reads). When optimizing the mismatch allowance, the need for greater sequencing throughput must be weighed against the need for lower error rates in the context of the particular application.

#### Primer trimming:

The final pre-processing step is sequence extraction, i.e., the isolation of the sequences of interest. This is achieved by removing conserved sequences (e.g., from library design) at the 5’ and 3’ regions, like priming sites, that could interfere with downstream analyses like clustering. Tools like CutAdapt^81^ and PANDASeq^82^ can perform this function with user-supplied sequences. Selecting extraction sites should be done with care, particularly on the 5’ end where the extraction site may set the reading frame during *in silico* translation. The 3’ end is also important, as it will determine where translation will be terminated if a stop codon was not included in the sequenced amplicon. It may be advantageous to remove the entire PCR primer region, since this region is not expected to be subject to mutation. However, if extracting from regions that were designed to be constant in the initial library but were subject to possible mutation, it is critical to optimize the extraction sequence and mismatch allowances; failure to do so can result in problems such as a large fraction of frame shifts or untranslatable sequences.

Library metrics should be collected at each step of the pre-processing pipeline to assess progress toward the goal of retaining the most reads at the highest quality as well as to quickly identify any errors of coding. Common metrics include average read quality scores, read length distributions, total read counts, unique read counts, and percentage of reads retained. By monitoring these metrics at each step, the pre-processing pipeline can be fine-tuned to optimize the final output. It should also be noted that pre-processing steps are not limited to those listed here; other methods like length filtering, head cropping, and contaminant filtering can be implemented as needed to further increase the quality of the final library.

Ultimately, the final indicator of successful pre-processing is the set of amino acid sequences produced by *in silico* translation of the pre-processed reads. These should have near-uniform length distributions at the expected length (or lengths), be consistent with the expected amino acid composition, and retain conserved or semi-conserved motifs and the overall structural framework, if any.

## 8. Summary and outlook

HTS has become a powerful tool for analyzing molecular evolution, and *in vitro* selection of mRNA-displayed peptides is a rising example of this trend. As the number of sequencing reads obtained by HTS technologies increases, pool compositions can be viewed with increasingly high resolution. Greater sequencing depth allows reduction of noise in estimation of some results (e.g., enrichment metrics), and, more importantly, it can capture qualitatively new information (e.g., delineating evolutionary pathways, including rare but important pathways; identifying low abundance but highly active sequences missed by low depth sequencing^40^; enabling quantitative analysis of fitness landscapes). In addition, if a traditional assay (e.g., measuring kinetic parameters) can be designed to give an output that can be measured by HTS^38, 85, 86^, the throughput of that assay can be increased by several orders of magnitude over classic biochemical methods. Because of this potential, conversion of traditional assays to HTS format is indeed a hotbed of research that shows no sign of slowing, as indicated by the rapid proliferation of methods relying on HTS.

As the affordability of HTS increases, future progress is expected in the field of molecular evolution. Experiments performed in the past, in which only a few variants were sequenced and tested for activity might now be the starting point to future studies. As long as samples for such experiments are still available, a kind of molecular archeology can be performed on the freezer samples. A notable subject of such study is the Lenski lab’s famous long-term evolutionary experiment (LTEE) on *E. coli*, which began in 1988 and has progressed through more than 60,000 generations^87–89^. Although the beginning of the LTEE predated HTS, freezer samples examined from early generations can reveal the emergence of new mutations, including new metabolic activities.

As HTS technology improves, it is interesting to consider whether greater sequencing depth is always desirable. While more information is undoubtedly obtained, greater computational time is also required to process the data, and in some samples, there is likely to be little overall benefit to greater sequencing depth. For example, the number of unique sequences (i.e., the pool complexity) in very diverse samples, such as the initial pool, probably exceeds the capacity of sequencing (i.e., 10^14^ different sequences); the benefit of 10^9^ reads compared to 10^8^ reads or even fewer is unclear. At the other extreme, for a highly converged pool of low complexity, such as would be derived from samples late in the selection, additional reads are also less useful; if the pool contains 100 unique sequences, the benefit of having 10^9^ vs. 10^8^ sequences is also marginal. Thus greater sequencing depth will become most useful for pools of intermediate diversity. Having said this, there are certain scenarios in which very deep sequencing of low and high complexity pools may be useful, such as if one intends to characterize the bias among *k*-mers of the synthesized pool^90, 91^ or if the frequency distribution of sequences in a highly converged pool is very uneven (i.e., some sequences of interest are present at very low abundance). Another special scenario that may require increasingly deep sequencing is the systematic exploration of fitness landscapes, in which all mutations at certain sites are investigated. If it is desirable to compare frequencies of each mutant before and after selection, then the number of reads that can be obtained from the initial pool becomes a critical parameter (i.e., a 20-fold increase in sequencing depth will allow one additional site to be explored by saturation mutagenesis).

At the same time, while HTS analysis offers important quantitative and qualitative advantages for the analysis of *in vitro* evolution experiments, care is required during each step of the analysis to ensure that the analysis itself does not bias the results (i.e., high quality data are preserved and artifacts are not introduced). Seemingly minor choices during data processing, such as number of errors tolerated in the adapter or primer sequence, or length of the sequence extracted, can have unexpectedly large effects on the quality and quantity of the resulting sequences; thus attention should be paid to any processing step that gives an unexpectedly low yield of passing sequences. Sequencing error is a frequent issue when studying evolutionary trajectories, since it is essential to distinguish between sequencing errors and true mutations. In our experience, experimental measures taken to reduce sequencing error rates (e.g., paired-end sequencing with stringent joining criteria, consensus sequencing, etc.) are usually worth their effort and expense in order to reduce uncertainty in data interpretation or the need for error correction strategies. It is also good practice to validate results obtained from HTS by classical biochemical assays whenever possible, to ensure the reliability of the results and expose any biases that may have been introduced by the HTS analysis itself. On a practical note, it can be useful to take advantage of rapid ‘micro’ or ‘nano’ low-output runs to generate a small preliminary data set to test the analysis pipeline as well as the quality of the input sample. For example, for the low complexity samples generated by *in vitro* evolution, such preliminary runs can uncover important but correctable sample issues.

As HTS instruments themselves decrease in cost and new instruments replace old ones, another interesting avenue for future research will be custom modification of the instruments themselves to achieve new goals. HTS technology combines miniaturization, massive parallelization, and highly sensitive detection – these features are assets to a number of potential applications. For example, an mRNA bound to the surface of a chip could be translated, assayed, and sequenced all at once^92^. Some sequencing technologies assay single molecules, and could probe not only nucleobases and epigenetic modifications but also other chemical varieties that could be of interest. In the next stage of technology exploration, it will become increasingly common not only to use HTS as a ‘black box’ that produces sequencing data, but to adopt and alter the hardware directly in the laboratory for new, ‘off-label’ applications.

## Figures and Tables

**Table 1.** Typical features of selection methods. The values features listed below represent estimates for the most common protocols for each selection method. Some deviations from those estimates are possible.

	Cell-based selections^a^	*In vitro* selections	
		Ribosome display	mRNA display
Library diversity	10^6^–10^9^	~10^13^	~10^13^
Genotype-phenotype connection	Non-covalent	Non-covalent	Covalent
Type of protein	Cell-compatible only	Any	Any
Temperature range	±5°C^b^	~4°C	0–100°C
Buffer conditions	Must be compatible with cell or phage integrity	High Mg^2+^, low T; must be compatible with ternary complex	Generally tolerant as long as compatible with chemical integrity of protein and RNA

a Selection parameters and conditions are limited to ensure compatibility with cell survival.

b Optimum temperature depends on the type of cell used. Phages may tolerate wider temperature ranges.

**Table 2.** Technical specifications of sequencing products.

Company	Platform	Run Time	Maximum Output	Maximum Read Length	Reads per run
Illumina Inc.	MiSeq	4 – 55 hrs	15 Gb	2 × 300bp	25 M/ per lane
	NextSeq	12–30 hrs	120 Gb	2 × 150bp	400 M / per lane
	HiSeq 3000	< 1 – 3.5 days	750 Gb	2 × 150bp	2.5 B /per lane
	HiSeq 4000	< 1 – 3.5 days	1,5 Tb	2 × 150bp	5 B / per lane
	HiSeq × Series	< 3 days	1,8 Tb	2 × 150bp	6 B /per flow cell
	NovaSeq 6000	~13 – 38 hrs	N/A	2 × 250bp	10 B/per lane
Pacific Biosciences Inc	PacBio RS II	0.5 – 4 hrs	1Gb	~10 – 15 kb	50 – 80 k
Life Technologies Corp.	Ion GeneStudio S5	4.5 –19 hrs	15 Gb	200 – 600 bp	2 – 130M
	Ion GeneStudio S5 Plus	3 – 20 hrs	30 Gb	200 – 600 bp	2 – 130M
	Ion GeneStudio S5 Prime	3–10 hrs	50 Gb	200 – 600 bp	2 – 130M
Sequencing by Oligo Ligation Detection	SOLiD 5500 W	10 days	120 Gb	2 × 50bp	1.2 B
	SOLiD 5500×1 W	10 days	240 Gb	2 × 50bp	2.4 B
Roche Inc.	454 GS FLX+	10–23 hrs	450 – 700 Mb	Up to 1 kb	1M
	454 GS Jr	10 hrs	35 Mb	400 bp	100 k
Oxford Nanopore	Flongle	1 min – 16 hrs	2 Gb	>2Mb	126 channels
	MinlON	1 min – 48 hrs	50 Gb		512 channels
	GridlON Mk1	1 min – 48 hrs	250 Gb		512×5 channels
	PromethlON 24	1 min – 72 hrs	5.2 Tb		24×3000 channels
	PromethlON 48	1 min – 72 hrs	10.5 Tb		48×3000 channels

Abbreviations *Mb*, *Gb* and *Tb* correspond to Megabytes, Gigabytes and Terabytes, respectively (for comparison, the human genome has 3×10^9^ bp or 3 Gb). Abbreviations *hrs* corresponds to hours, *bp* to base pair, *kb* to kilobase, *M* and *B* to millions and billions.

**Table 3.** Comparing sequencing technologies.

	Advantages	Disadvantages	Library amplification	Sequencing technology
Illumina Inc.	Large user base platform Low cost per base High coverage (high output)	Short reads	Bridge-PCR on flow cell surface	Reversible terminator sequencing by synthesis
Pacific Biosciences Inc	Very long reads (> 1 kb) Short run time Low reagents cost	High basal error rate Low output	NA	Single-molecule, real-time DNA sequencing by synthesis
Life Technologies Corp.	High coverage Longer reads	Lower output	PCR on FlowChip surface	Polymerase synthesis
Sequencing by Oligo Ligation Detection	Low cost per base Low reagents cost Inherent error correction (two-base encoding)	Short reads Long run time	Emulsion PCR	Sequencing by ligation
Roche Inc.	Longer reads Short run times High coverage	Higher cost per base High reagents cost High error rates in homopolymer repeats	Emulsion PCR on microbeads	Pyrosequencing
Oxford Nanopore	Very long reads Customization	High error rate Difficult to design multiple parallel pores	NA	Nanopore exonuclease sequencing

Basic advantages and disadvantages of different sequencing platforms and the sequencing technology or chemistry they use. *NA* means not applicable.
